# Special Issue: MicroRNA Regulation in Health and Disease

**DOI:** 10.3390/genes10060457

**Published:** 2019-06-15

**Authors:** Subbaya Subramanian, Clifford J. Steer

**Affiliations:** 1 Department of Surgery, University of Minnesota Medical School, Minneapolis, MN 55455, USA; 2 Department of Medicine, University of Minnesota Medical School, Minneapolis, MN 55455, USA; 3 Department of Genetics, Cell Biology and Development, University of Minnesota Medical School, Minneapolis, MN 55455, USA

Our understanding of non-coding RNA has significantly changed based on recent advances in genomics and molecular biology, and their role is recognized to include far more than a link between the sequence of DNA and synthesized proteins. MicroRNAs (miRNAs) are small regulatory RNAs that play a crucial role in posttranscriptional gene regulation. Greater than 2500 miRNAs have been identified and catalogued in humans and many of them are conserved in other species [1]. miRNAs are implicated in almost every facet of fundamental cellular functions including development, senescence and disease. The past decade has experienced a remarkable increase in the understanding of miRNA biogenesis, their target genes, miRNA biomarkers and potential therapeutics for a growing number of disease conditions. RNA-based markers and therapeutics have a potentially significant clinical impact, and many of the miRNA-based therapies are at various stages of application and human clinical trial [2].

As background, non-coding RNAs are divided into (i) transcription RNAs (including both tRNA and rRNA); (ii) small RNAs, which are further subdivided into siRNAs, miRNAs, snoRNAs, and snRNAs; and (iii) most recently, long non-coding RNAs, which are now know to transcribe short peptides [3]. MicroRNAs are single-stranded non-coding RNAs that are typically 18–25 nucleotides (nts) in length and are best known for their role in the post-transcriptional regulation of gene expression. They are the most abundant class of small endogenous non-protein coding RNAs, and make up one of the largest well-conserved gene families found among viruses, plants and animals. The majority of miRNA sequences in humans are typically transcribed by introns of non-coding and coding transcripts, with few transcribed by exonic regions.

MicroRNA genes are typically transcribed by polymerase II or III and generate primary miRNAs (pri-miRNAs), which can contain sequences for multiple miRNAs and be hundreds of nts in length. These structures are then processed and cleaved by Drosha-DGCR8 complex, resulting in the formation of a hairpin-shaped stem-loop structure, which is known as the precursor miRNA (pre-miRNA) and typically around 70 nts in length. The pre-miRNA is exported outside of the nucleus primarily by exportin 5. Further processing takes place in the cytoplasm by Dicer1-TARBP2, which is an RNase III enzyme, resulting in a two-stranded duplex of miRNA-miRNA*. Typically, it is 18–25 nt long, with one strand designated the guide strand and the other as the passenger strand. Finally, the guide strand is incorporated into the RNA-induced silencing complex (RISC), which is a large multiprotein miRNA ribonucleoprotein complex that is the effector compound in modulating target gene transcription. Alternative pathways have been described that are Drosha-DGCR8 independent as well as Dicer-independent, and are likely to greatly advance our understanding of miRNA biogenesis and involvement in conditions of health and disease.

Regulatory interactions between miRNA and other noncoding RNAs, including long noncoding RNAs (lncRNA), and circular RNA (circRNA) are now known to determine the cellular functional status and phenotype. MicroRNAs use seed sequences (6–8 bases long) to bind microRNA Response Elements (MREs) located on their interacting partners, primarily at the 3’UTR of coding transcripts [4]. However, it is possible that the frequency of MREs in the entire transcriptome of a given cell contributes to the dynamic gene regulatory process by acting as a sponge for mature miRNAs, thus regulating their functional availability. Thus, the coding and noncoding transcripts sharing common MRE sites compete with each other and define the gene expression profile of a given cell. These competing transcripts are collectively called competing endogenous RNAs [5]. Thus, gene expression regulation is a complex process involving the dynamic interactions between miRNA-mRNA-lncRNA-circRNA. This complexity is increased multifold when these interacting partners are exchanged between cells via extracellular vesicles. Recognizing this intricate system will significantly aid in our understanding of the health and disease process. We are beginning to study disease pathogenesis at the cellular, organ, and whole body levels; and the gut microbiota is increasingly recognized as a crucial player [6]. It is time to acknowledge each of these players as they take center stage in maintaining homeostasis and the normal physiological functioning of an organism.

There are still significant gaps in understanding the complex regulatory mechanisms of miRNAs; however, the field is advancing rapidly. Further, it has been shown that a number of nuclear receptors are involved in the transcriptional regulation of miRNA expression, including the small heterodimer partner (SHP) and farnesoid X receptor (FXR). In general, miRNAs are detected as (i) extracellular circulating miRNA bound to different lipoproteins; (ii) part of a non-membrane ribonucleoprotein complex associated with Argonaut proteins; and (iii) contained in exosomes as extracellular vesicles, where they act as nano-sized transporters involved in the communication between neighboring cells. The recent finding that miRNAs are also transported from one cell to another via tunneling nanotubes underscores their importance in maintaining communication among all cell types, including those associated with cancer.

This Special Issue of *Genes*, entitled “MicroRNA Regulation in Health and Disease” consists of a series of articles spanning the clinical realm from colorectal cancer to pulmonary fibrosis. However, we begin with a research article by Liu et al. who reported for the first time the existence of complemented palindromic small RNAs (cpsRNAs) from SARS coronavirus, and propose that cpsRNAs and palindromic small RNAs (psRNAs) constitute a novel class of small RNAs [7]. Such a discovery of cpsRNAs could pave a way to find novel markers for pathogen detection and to reveal the mechanisms underlying infection or pathogenesis from a different perspective. In a study titled, “A Two-Cohort RNA-seq Study Reveals Changes in Endometrial and Blood miRNome in Fertile and Infertile Women”, Rekker et al. compared mid-secretory phase samples between fertile and infertile women [8]. The study revealed 21 differentially expressed miRNAs from the endometrium and one from blood samples. Among the novel miRNAs, chr2_4401 was validated and showed upregulation in the mid-secretory endometrium. In addition to the novel findings, the authors confirmed the involvement of miR-30 and miR-200 family members in mid-secretory endometrial functions. Hueso et al. elegantly showed in their article that an exonic switch regulates the differential accession of microRNAs to the *Cd34* transcript in atherosclerosis progression [9]. Further, they proposed a new mechanism of miRNA action, linked to a cryptic splicing site in the target-host gene, that would regulate the differential accession of miRNAs to their cognate binding sites.

Li et al. studied the role of miRNA-106a-5p in inhibiting C2C12 Myogenesis via targeting PIK3R1 and modulating PI3K/AKT signaling [10]. Their results showed that miR-106a-5p was elevated in aged muscles and dexamethasone (DEX)-treated myotubes. The up-regulation of miR-106a-5p significantly reduced the diameters of myotubes accompanied by increased levels of muscular atrophy genes and decreased PI3K/AKT activities. Finally, miR-106a-5p was demonstrated to directly bind to the 3’-UTR of PIK3R1, thus, repressing PI3K/AKT signaling. The microbiome appears to interact and perhaps influence an unlimited number of metabolic processes in health and disease. In their article, Yuan et al. postulate that the altered nutrient composition and miRNA expression in colorectal cancer (CRC) microenvironment selectively exerts pressure on the surrounding microbiota, leading to alterations in its composition [11]. Further, the authors present a detailed overview of the current understanding of the role of miRNAs in mediating host-microbiota interactions in CRC. “Single Nucleotide Polymorphisms in *MIR143* Contribute to Protection Against Non-Hodgkin Lymphoma (NHL) in Caucasian Populations” by Bradshaw et al. is first to report a correlation between miRSNPs in *miR-143* and a reduced risk of NHL in Caucasians [12]. Further, it is supported by significant SNPs in high linkage disequilibrium (LD) in a large European NHL genome-wide association study (GWAS) meta-analysis. Axmann et al. compared the miRNA profiles in serum and lipoprotein particles of healthy individuals with those of patients with uremia [13]. They observed a significant increase in levels of cellular miRNA level using reconstituted high-density lipoprotein (HDL) particles artificially loaded with miRNA, whereas incubation with native HDL particles yielded no measurable effect. Based on the results, the authors concluded that there was no relevant effect of lipoprotein-particle-mediated miRNA-transfer under in vivo conditions though the miRNA profile of lipoprotein particles can be used as a diagnostic marker.

Mullenbrock et al., carried out an elegant systems analysis transcriptomic and proteomic study on the potential role of miRNAs in pulmonary fibrosis [14]. They specifically targeted fibroblasts and myofibroblasts as the key effector cells responsible for the excessive extracellular matrix (ECM) deposition and fibrosis progression in both idiopathic pulmonary fibrosis (IPF) and systemic sclerosis (SSc) patient lungs. The comprehensive analyses of mRNA, miRNA, and matrisome proteomic profiles in IPF and SSc lung fibroblasts revealed robust fibrotic signatures at both the gene and protein expression levels and identified novel fibrogenesis-associated miRNAs whose aberrant downregulation in disease fibroblasts likely contributes to their fibrotic and ECM gene expression. Somatostatin (SST) analogues were used to control the proliferation and symptoms of neuroendocrine tumors (NETs) in an article by Døssing et al., entitled “Somatostatin Analogue Treatment Primarily Induce miRNA Expression Changes and Up-Regulates Growth Inhibitory miR-7 and miR-148a in Neuroendocrine Cells” [15]. Two miRNAs which were highly induced by SST analogues, miR-7 and miR-148a, were shown to inhibit the proliferation of NCI-H727 and CNDT2 cells. SST analogues also produced a general up-regulation of the let-7 family members. SST analogues controlled and induced distinct miRNA expression patterns among which miR-7 and miR-148a both have growth inhibitory properties.

As FDA-approved small RNA drugs begin to enter the arena of clinical medicine, it is critical to expand both preclinical and clinical research studies for miRNAs. A growing number of reports suggest a significant utility of miRNAs as biomarkers for pathogenic conditions, modulators of drug resistance, and/or as drugs for medical intervention in almost all human health conditions. The pleiotropic nature of this class of nonprotein-coding RNAs makes them particularly attractive drug targets for diseases with a multifactorial origin and few, if any, available treatments. The landscape of both diagnostic and interventional medicine will arguably continue to evolve as candidate miRNAs pass successfully through phase 2 and 3 clinical trials. In this special issue of *Genes*, we provide a series of articles that highlight microRNAs as diagnostic, predictive and therapeutic agents for human disease. The development of bioinformatics programs to identify miRNA-binding sites in target genes and their corresponding biological pathways, along with an expanding platform of in vitro and in vivo preclinical research models, has propelled miRNAs into clinical medicine. The first siRNA human trial was conducted in 2004 and, in 2018, the first siRNA drug was approved, paving the way for a class of miRNA transcripts whose active investigation began only a little more than 15 years ago. The future of human miRNA clinical trials is absolutely guaranteed and that time has arrived.

The development of miRNA diagnostics and therapeutics is an exciting and potentially new frontier in treating diseases for which few treatment options exist. We believe and hope that this Special Issue of *Genes* will be an important resource for a wide variety of audiences, including students at all levels, and established investigators who are interested in contributing to the remarkable and ever-expanding field of microRNAs in health and disease.
